# A novel non-invasive method of measuring microcirculatory perfusion and blood velocity in infants: a pilot study

**DOI:** 10.1038/s41598-022-10911-8

**Published:** 2022-05-06

**Authors:** Norani H. Gangaram-Panday, Louwrina H. te Nijenhuis, Ilya Fine, Irwin K. M. Reiss, Willem van Weteringen

**Affiliations:** 1 Department of Pediatrics, Division of Neonatology, Erasmus MC Sophia Children’s Hospital, University Medical Center Rotterdam, Rotterdam, The Netherlands; 2 Elfi-Tech Ltd., Rehovot, Israel

**Keywords:** Blood flow, Paediatric research, Diode lasers, Neonatology

## Abstract

Current haemodynamic monitoring is mainly aimed at the macrocirculation. Multiple studies have demonstrated the importance of the microcirculation in relation to the patient’s condition and impact of treatment strategies. However, continuous monitoring of the microcirculation is not yet possible in the neonatal field. A novel dynamic light scattering (DLS) sensor technology for continuous monitoring of the microcirculation was investigated in the neonatal population. Thirty-one haemodynamically stable infants were included. Sequential measurements at the forehead, upper extremity, thorax, abdomen and lower extremity were conducted with the DLS sensor. For analyses stable measurements were selected. The DLS parameters, total blood flow (TBF) and relative blood velocity (RBV), were compared between measurement locations. Changes in relative haemodynamic indices (relHIs), indicating the distribution of blood flow in the microcirculatory blood vessels, were associated with heart rate decelerations. Measurements performed at the forehead had significantly lower TBF levels, compared to measurements at other locations. Early changes in relHIs around a heart rate deceleration were recorded a median (IQR) of 22.0 (13.5–27.0) s before the onset. Measurement of the currently unavailable parameters TBF, RBV and relHIs is possible with DLS technology. Validation of the DLS technology is needed for clinical implementation.

## Introduction

Current methods for continuous monitoring of haemodynamics are mainly aimed at the macrovascular system, varying from heart rate monitoring to measurement of blood pressure, central venous pressure, pulse index and capillary refill time. Several studies have shown that the microvascular system is of equal importance when it comes to treatment strategies in critically ill patients, in whom the relation between tissue perfusion and systemic haemodynamics is lost^1–7^. This link between the macrovascular and microvascular state is called haemodynamic coherence^4^. Whenever treatment strategies result in improvement of macrocirculatory parameters but do not improve the microcirculation, further deterioration of the microcirculation can occur with potentially fatal outcome^2,4,8^. Continuous monitoring of the microcirculation to evaluate tissue perfusion, oxygen delivery and therapeutic interventions is thus essential for full assessment of the haemodynamic system.

Despite the vast interest in measurement of the microcirculation, monitoring techniques are mostly experimental. Many technologies, which are currently used for clinical care, provide indirect information on the blood perfusion, such as photoplethysmography (PPG) and transcutaneous oxygen monitoring^9–11^. A variety of technologies for the direct measurement of blood flow or velocity, such as Doppler ultrasound, laser Doppler flowmetry, video microscopy and other microscopy techniques, including dark-field imaging^12–17^, have been evaluated in different patient populations^4,5,18^. An abnormal microcirculatory flow has been related to increased risks of morbidity and mortality^2,19–27^. Treatment with vasoactive drugs, such as dobutamine, showed an improvement of the microcirculation in critically ill patients^28^. However, comparable results were not reported with therapeutic strategies aimed at the macrovascular system, such as norepinephrine^29–31^. Although several technologies for monitoring the microcirculation have been developed, their clinical value remains limited due to often intermittent measurements, absence of miniaturization and moderate ease of use. Early detection of alterations in the microcirculation is currently not feasible in neonatal clinical care.

Dynamic light scattering (DLS) is a new technology for the assessment of the haemodynamic system in the skin microcirculation. Tissue and blood vessels are illuminated with near-infrared laser light, which scatters from randomly moving red blood cells (RBCs) onto the detectors. The measured intensity over time depends on the relative velocity of the scattering particles. In comparison, laser Doppler flowmetry utilizes the Doppler shift, which is the change in light wavelength caused by moving particles. DLS technology has the advantage of providing information on the motion properties of the individual particles. The miniaturization of this technology makes it suitable for non-invasive monitoring, even in premature neonates^32^. In addition to the heart rate, DLS-based devices can continuously measure the perfusion (total blood flow (TBF)), the relative blood velocity (RBV) and relative haemodynamic indices (relHIs)^33,34^. While TBF is a blood volume-dependent parameter, RBV and relHIs depend on microcirculatory shear rates that are comparable between patients. The relationship between systemic haemodynamics and the microcirculation can be investigated with the RBV. In neonates, changes in cardiac output (CO) originate mainly from changes in heart rate, due to a limited capacity to increase myocardial contractility and thus stroke volume^35^. This specific property of the neonatal haemodynamic system allows monitoring of the consequences of macrocirculatory changes for the microcirculation. This study investigated microcirculatory parameters, measured with DLS technology, and their relation to the macrocirculation in the neonatal population. Based on the concept of haemodynamic coherence^4,36,37^, we hypothesized that changes in DLS microcirculatory parameters could precede neonatal heart rate decelerations. Depending on the origin of the events, such as a decrease in microvascular resistance, haemodynamic changes could first be visible in the most peripheral blood vessels.

## Methods

### Study design

**Figure 1:**
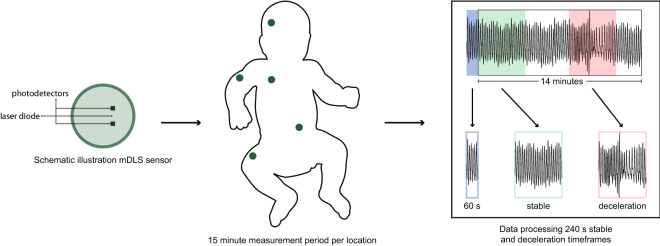
Schematic representation of the DLS sensor and the study protocol. *s* seconds.

This study was an in-depth analysis of a single-centre prospective observational cohort study aimed at the assessment of the DLS technology in infants (gestational age (GA) ≥ 26 weeks) admitted to the neonatal intensive care unit (NICU)^32^. Haemodynamically stable infants without severe incidents, invasive ventilation or not diagnosed with neonatal sepsis, were included in this study after informed consent was obtained from parents or legal guardians. Infants were included at any time during their admission. Stability of the DLS measurement was determined based on the percentual difference between heart rate measured with electrocardiography (ECG) and DLS, compliant with the IEC 60601-2-27:2011 standard^38^. For this in-depth study, stable infants were included with accurate norm-compliant DLS measurements at the forehead, upper extremity, thorax, abdomen and/or lower extremity, excluding the first 60 s of DLS measurements for each 15 min measurement period per location. The first 60 s were excluded, because of the expected signal interference due to movement artefacts after initial sensor placement (Fig. 1). Data on patient characteristics and high frequency physiological data, together with the DLS measurements, were collected on the day of measurement. The medical ethical board of Erasmus MC approved this study (MEC-2017-059). The study was performed in accordance with the relevant guidelines and regulations.

### DLS measurement

Dynamic light scattering technology, miniaturised in a 10 mm diameter sensor, allows continuous non-invasive monitoring of the haemodynamic system and the microcirculation. The mDLS™ sensor (software v3.81, data collection system PC GUI v1.0.9.5, Elfi-Tech Ltd., Rehovot, Israel) was used as an investigational medical device. Using a class I vertical-cavity surface-emitting laser (peak energy 0.85 mW) with a wavelength of 852.4 ± 2 nm, and an optoelectronic detection system, both pulsatile and non-pulsatile signal components can be detected. The penetration depth of human skin at this wavelength is approximately 1.3 mm. Light emitted by the sensor scatters off moving red blood cells into the geometrically aligned detectors, creating a time-dependent speckle pattern. From this pattern, the velocity and number of particles can be extracted and multiplied, indicating perfusion or the total blood flow (TBF) in the skin. The total blood flow is subject to patient-specific characteristics and presented in arbitrary units (AU). The relative blood velocity (RBV), which can be detected in a range from 0 to approximately 9000 Hz^−1^, is independent of the number of particles and relates to laser Doppler output. Both TBF and RBV were logged at a 1 Hz rate. The sensor was attached to the skin using a double-sided skin adhesive (LEA Medizintechnik GmbH, Giessen, Germany). There was no direct lighting on the sensor during measurements.

### Relative haemodynamic indices

**Figure 2:**
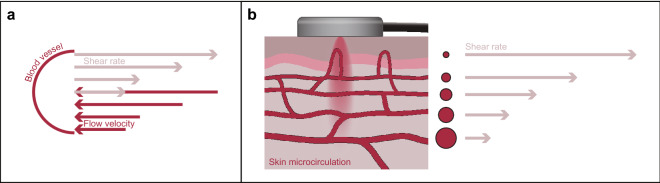
(**a**) The concept of vascular flow velocity and shear rates, showing high flow velocity at the centre of the blood vessel and the lowest flow velocity at the blood vessel wall. An inverse relation is shown for shear rate. (**b**) Schematic representation of a DLS sensor on the skin and the inverse relation between blood vessel diameter and the measured shear rate.

When laminar flow predominates random RBC movement, the DLS technology has the ability to process the signal and to distinguish flow in different types of microcirculatory skin blood vessels. The values of haemodynamic indices (HIs) are derived from the blood flow pattern of the mDLS sensor and characterize the shear rate, which is an important parameter of the microcirculation^39^. The shear rate is defined as the gradient of the velocity profile inside the blood vessels, with a maximal flow velocity at the centre of the blood vessel and a maximal shear rate at the vascular wall. In any given vessel, a higher blood flow velocity results in a greater shear rate (Fig. 2). In addition, the shear force and shear rate depend on blood viscosity and resistance of the vascular endothelium to movement of erythrocytes adjacent to the vessel wall. Hence, HIs depend on the type of blood vessel, the vascular tone, resistance to the flow, and the blood flow velocity. The lowest shear rates are predominantly present in the larger vessels of the venous and capillary circulation. Under normal physiological flow conditions, the shear rate increases from about 10 s^−1^ in veins to about 2000 s^−1^ in the smallest arteries^40^.

Regulatory mechanisms can change the distribution between different blood vessel types, depending on the neurological or metabolic processes taking place. As a result, either or both the blood volume and velocity may increase. To discriminate such changes, relative HIs (relHIs) were developed to characterize the relative contribution of blood flow of a specific range of shear rates relative to all others. This can be clinically translated to the relative distribution of blood vessel sizes. The relHIs were calculated with a 100 Hz sampling rate and are presented in AU, ranging between 0 and 1.

### Heart rate, oxygen saturation and body temperature

ECG (Dräger M540, Dräger Medical GmbH, Lübeck, Germany) and pulse oximetry (Masimo SET Radical pulse oximeter, Masimo Corp., Irvine, CA, USA) were used for heart rate detection and oxygen saturation monitoring (SpO_2_), respectively (software vVG3). Both parameters were logged at a 1 Hz rate. Body temperature was recorded using a temperature probe placed underneath the infant or within the diaper.

### Definitions

Haemodynamic stability was defined as infants without severe bradycardias, apnoeas and desaturations during nursing, physical examination and kangaroo care. Based on the hospital guideline, a moderate to severe patent ductus arteriosus (PDA) was defined as a ductus arteriosus diameter larger than 1.5 mm on ultrasound, performed by a paediatric cardiologist during standard care. This definition was considered to be a haemodynamically significant PDA. Heart rate decelerations were defined as a reduction in heart rate of 30 beats per minute (bpm) from the calculated median heart rate per patient.

### Signal processing

From all DLS measurement data with IEC 60601-2-27:2011-compliant stability, the first 240 stable data points without heart rate decelerations were included in a 240 s timeframe for analysis of the relationship between the microcirculation and macrocirculation. In addition, if a heart rate deceleration occurred during a DLS measurement, this moment was selected, including a 240 s timeframe surrounding the start of the heart rate deceleration. There was no overlap between the two timeframes (Fig. 1). For each heart rate deceleration, the exact start times of visible changes in ECG heart rate, SpO_2_ and relHIs were marked by two researchers. The end point was marked in both the ECG heart rate and relHI signal. The area under the curve (AUC) of the measured relHI changes during a heart rate deceleration was calculated. The relHI value marked as deceleration onset was used as the baseline and the AUC was defined as the cumulative absolute relHI deviation from the baseline within the marked time period.

### Statistical analyses

Patient demographics are reported as median (interquartile range (IQR)) or number (n) (%). To compare TBF and RBV levels at all measurement locations, means were calculated of all IEC 60601-2-27:2011 norm-compliant DLS measurement data at each location per patient and are presented as mean with the standard deviation (SD). A paired t-test with a Bonferroni correction was applied to test differences between measurement locations for which significance was set at a *p*-value < 0.005. A Wilcoxon rank sum test was used to investigate the differences between infants with a haemodynamically significant PDA and infants without PDA and between infants receiving phototherapy and infants who did not receive any phototherapy during measurements.

To determine the relation between the heart rate and RBV, a linear mixed model was fitted on stable data without a heart rate deceleration. In the random effects structure time, quadratic time, cubic time and subjects were included to adjust for repeated measures. GA at measurement, measurement location, postnatal age and PDA, together with the ECG heart rate, were included in the fixed effects structure and assessed using backward selection. Interactions between GA at measurement and heart rate, and between measurement locations and heart rate were included in the fixed effects structure. In addition, non-linearity in the relation between heart rate and RBV was assessed using splines with boundary knots at the 5th and 95th percentile. Analysis was performed using the nlme package for R^41^.

Differences between the mean and SD of stable relHI values and relHI values during a heart rate deceleration were tested using a Wilcoxon signed rank test. All analyses were performed in R (version 4.0.0, Inc., Boston, MA, USA) and MATLAB (version R2020a, The Mathworks, Inc., Natick, MA, USA). Missing values were omitted as they were assumed to be missing completely at random. A *p*-value of < 0.05 was considered significant.

## Results

### General characteristics

**Figure 3:**
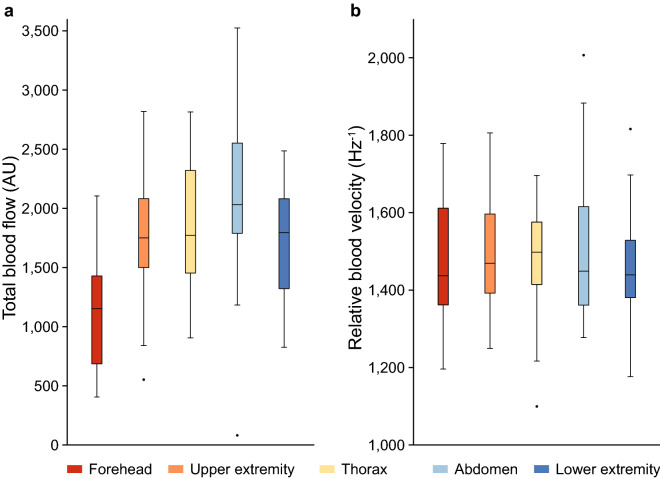
Boxplots of DLS parameters perfusion (total blood flow) (**a**) and relative blood velocity (**b**) per measurement location. Significance was found only between perfusion measurements on the forehead and other measurement sites. *AU* arbitrary unit.

A total of 31 infants were included for analyses of perfusion and blood velocity (Table 1). Nineteen heart rate decelerations were recorded in fifteen infants during the study period. In addition, three infants were diagnosed with a haemodynamically significant PDA, for which treatment was started. The mean TBF and RBV at each measurement location are shown in Fig. 3 and Supplementary Table S1. A significant difference was found only between the TBF measured at the forehead and at other measurement locations. No significant differences were reported between DLS measurements at the left upper extremity in infants with a PDA (n = 3) and at either upper extremity in infants without a PDA (n = 25), for the measured TBF (significant PDA 1481 (855–2035) AU vs. no PDA 1862 (1560–2095) AU; *p* = 0.373) and RBV (significant PDA 1509 (1467–1549) Hz^−1^ vs. no PDA 1437 (1364–1635) Hz^−1^; *p* = 0.603). Three infants with a suspected PDA were not included in this analysis because a cardiac ultrasound was not performed. Differences in measurements were found between infants with (n = 8) and without (n = 23) phototherapy in TBF levels (1895 (1748–2067) AU vs. 1654 (1414–1812) AU; *p* = 0.008), but not in RBV levels (1447 (1380–1487) Hz^−1^ vs. 1498 (1444–1514) Hz^−1^; *p* = 0.142).

**Table 1:** Patient baseline characteristics.

	Total (n = 31)
GA at birth (weeks)	30 3/7 (24 6/7–40 2/7)
Birth weight (g)	1283 (575–4000)
SGA	13 (41.9)
APGAR score at 1 min	6 (4–8)
APGAR score at 5 min	8 (7–9)
Arterial umbilical cord pH	7.30 (7.25–7.33)
Gender (male)	15 (48.4)
Multiple birth	11 (35.4)
GA at measurement (weeks)	31 6/7 (28 4/7–42 2/7)
Weight at measurement (g)	1400 (910–4200)
Postnatal age (days)	6 (3–18)
Admission survival	29 (93.5)
**Ventilation**	
Non-invasive	24 (77.4)
No ventilation	7 (22.6)
**Environment**	
Incubator	18 (58.1)
Heat mattress	13 (41.9)
Incubator temperature (°C)	31.0 (29.5–32.1)
Phototherapy	8 (25.8)
**Body temperature (°C)**	
Incubator*	36.8 (36.5–37.1)
Heat mattress**	36.6 (36.6–37.2)
Heart rate (bpm)	153 (146–161)

GA at birth, birth weight, GA at measurement and weight at measurement are reported as median (range). All other data is presented as median (IQR) or number (%). *GA* gestational age, *SGA* small for gestational age. Missing values *(n = 1) and **(n = 2).

### Relationship between RBV and HR

**Figure 4:**
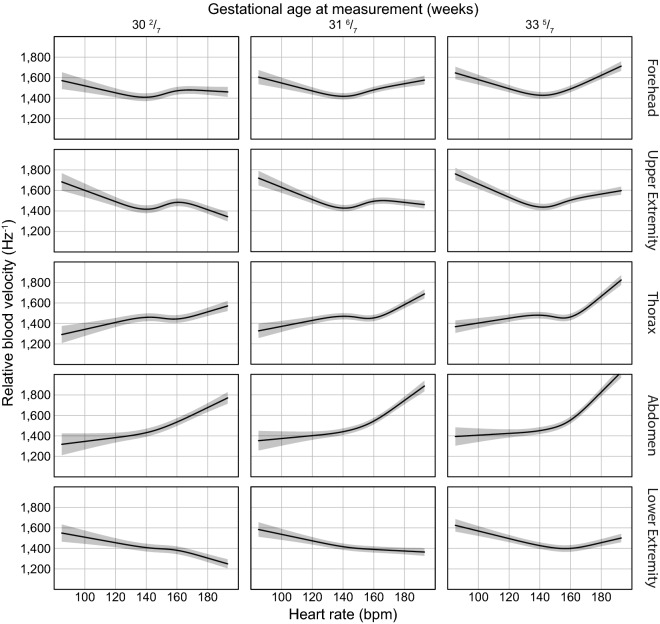
Effect plots illustrating the estimate and 95% confidence intervals of the relation between relative blood velocity and heart rate, gestational age at measurement and measurement locations. The nonlinear relation of heart rate and interactions between gestational age at measurement (25th, 50th and 75th percentiles) and location are presented. *Bpm* beats per minute.

A total of 31,200 paired data points, measured during a stable period, were included from all infants at the forehead (n = 7440), upper extremity (n = 7440), thorax (n = 6720), abdomen (n = 2880) and lower extremity (n = 6720). The relationship between the RBV and heart rate measured with ECG is presented in Fig. 4, showing effect plots including 95% confidence intervals of the estimates. Significant relations were found between the RBV and the heart rate, the GA at measurement and the measurement location, for which they were included as fixed effects in the linear mixed model. Nonlinearity of ECG heart rate was included in the model. The interaction between heart rate and the GA at measurement, and the interaction between the measurement location and the heart rate were also included (Supplementary Table S2). Noticeable was the shift towards a V-shaped relationship between the heart rate and the RBV with increasing GA at measurement for all locations, indicating an increase of the RBV with both lower and higher heart rates.

### Distribution of the relative haemodynamic indices

**Figure 5:**
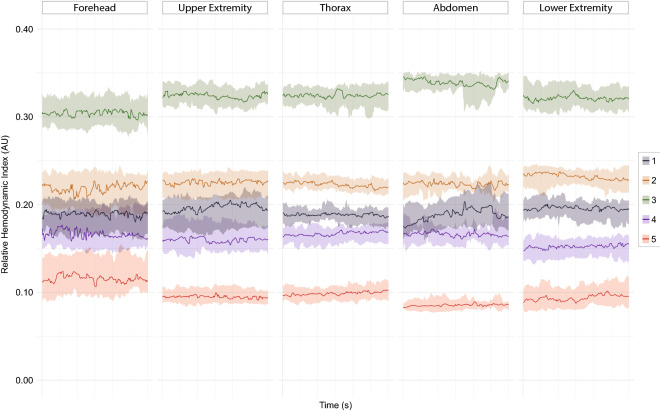
Plots of the medians and interquartile ranges of the relative haemodynamic indices at all measurement locations, illustrating the smallest vessels (1), small vessels (2), medium-sized vessels (3), large vessels (4) and largest vessels (5) in the microcirculation. *AU* arbitrary unit, *s* seconds.

Stable data of all 31 infants were included to illustrate the distribution of the relHIs in the microcirculation (Fig. 5). Median values of relHIs indicating the smallest microcirculatory blood vessels (1 and 2) were similar for every location, while the relHIs indicating the medium-sized (3) and largest (5) microcirculatory blood vessels, showed strong variation among measurement locations. In measurements at the lower extremities, the flow in the medium-sized (4) blood vessels was lower when compared to measurements at other locations. This location showed a distinct separation of the relHIs, while at other locations the distribution of the relHIs was less distinguishable.

### Effect of heart rate decelerations on the relative haemodynamic indices

**Figure 6:**
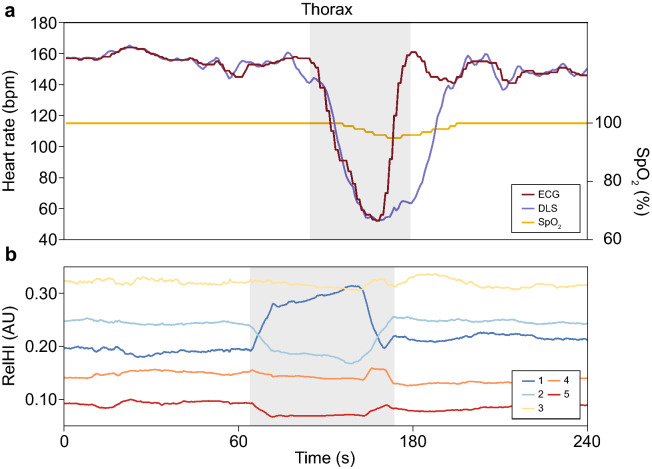
An example of a 240 s time interval of a bradycardic event. (**a**) ECG heart rate, DLS heart rate and SpO_2_ measurements are shown. The delay in the recovery of the DLS heart rate, was caused by movement of the infant introduced by the nurses. (**b**) Relative haemodynamic indices are presented; smallest vessels (1), small vessels (2), medium-sized vessels (3), large vessels (4) and largest vessels (5). *RelHI* relative haemodynamic index, *bpm* beats per minute, *s* seconds, *ECG* electrocardiography, *DLS* dynamic light scattering, *SpO*_*2*_ oxygen saturation measured with pulse oximetry.

At all measurement locations, a change in the relHIs was recorded before a heart rate deceleration (n = 19) was detected in heart rate measurements with ECG (22.0 (13.5–27.0) s). Desaturations were recorded after the onset of the heart rate deceleration (9.0 (5.0–15.3) s). Figure 6 illustrates an example of a bradycardic event in a preterm infant. Heart rate decelerations showed a median (range) drop of 42 (30–111) bpm. Signal changes were primarily recorded in the medium-sized (4) blood vessels. The largest change in relHIs, presented as the AUC, was found in the smallest (1) blood vessels (0.698 (0.254–1.288) s^−1^). When compared to a stable period, only the mean relHI of the smallest (1) blood vessels changed significantly during a heart rate deceleration (Table 2). The SDs of all relHIs changed significantly during a heart rate deceleration. The largest change in SD was reported in the small (1 and 2) and largest (5) microcirculatory blood vessels (stable 0.004 (0.002–0.007) vs heart rate decelerations 0.009 (0.007–0.010); *p* = 0.001, stable 0.003 (0.002–0.005) vs heart rate decelerations 0.006 (0.005–0.008); *p* < 0.001 and stable 0.002 (0.001–0.003) vs heart rate decelerations 0.005 (0.004–0.010); *p* = 0.005, respectively) (Table 2).

**Table 2:** Relative haemodynamic indices in a stable and heart rate decelerations.

relHIs	Stable		Heart rate deceleration		*p*-value Mean	*p*-value SD	Heart rate deceleration
n = 19	Mean	SD	Mean	SD			AUC (AU*s)
1	0.190 (0.170–0.201)	0.004 (0.002–0.007)	0.195 (0.180–0.206)	0.009 (0.007–0.014)	0.049	0.001	0.698 (0.254–1.288)
2	0.230 (0.225–0.239)	0.003 (0.002–0.005)	0.229 (0.223–0.236)	0.006 (0.005–0.008)	0.617	< 0.001	0.336 (0.196–0.732)
3	0.324 (0.316–0.344)	0.004 (0.003–0.006)	0.315 (0.306–0.339)	0.008 (0.006–0.011)	0.980	0.002	0.472 (0.268–1.357)
4	0.167 (0.148–0.166)	0.003 (0.002–0.004)	0.158 (0.145–0.170)	0.007 (0.004–0.010)	0.710	0.004	0.439 (0.303–0.652)
5	0.094 (0.082–0.102)	0.002 (0.001–0.003)	0.100 (0.090–0.105)	0.005 (0.004–0.010)	0.065	0.005	0.591 (0.127–1.210)

Median (IQR) values of the mean and standard deviation per infant during a stable and heart rate deceleration phase in the (1) smallest vessels, (2) small vessels, (3) medium-sized vessels, (4) large vessels and (5) largest vessels. The relHIs are presented in arbitrary units (AU) and the AUC in AU*s. *relHIs* relative haemodynamic indices, *IQR* interquartile range, *SD* standard deviation, *AUC* area under the curve.

## Discussion

This is the first study investigating the blood flow parameters TBF, RBV and relHIs, non-invasively measured with a DLS sensor in the neonatal population. TBF was significantly lower on the forehead when compared to other measurement locations. A relationship was found between RBV values and the measurement location, GA at measurement and the heart rate. At a higher GA at measurement, both a lower and higher heart rate were related to a high RBV. Changes in the distribution of relHIs were associated with changes in heart rate, and were present a median of 22 s before heart rate decelerations were registered on the ECG. DLS technology can non-invasively measure haemodynamic parameters that are currently clinically unavailable and can potentially provide essential information on the microcirculatory status in the neonatal population.

Investigated infants were haemodynamically stable, so haemodynamic coherence between macrocirculation and microcirculation can be assumed to have been intact, which is confirmed by the results of this study. Changes in the macrocirculation were clearly visible in measurements on the microcirculation. In neonates, measurements of blood flow or CO are currently not available for continuous haemodynamic monitoring. The main clinical haemodynamic parameters are heart rate and blood pressure, which provide limited information on tissue perfusion. Heart rate and blood pressure levels in infants have not been related to the resulting microcirculatory blood flow. Most interventions are aimed at the macrocirculation, for example the administrations of inotropic agents to increase arterial blood pressure. Despite adequate inotropic support, deterioration of the microcirculation and subsequent loss of haemodynamic coherence can go undetected. This study demonstrates that in the investigation of the relation between blood pressure and alterations in skin and organ blood flow or RBV in infants, the effects of patient-specific characteristics, such as gestational and postnatal age, should be taken into account.

This study describes the relation between heart rate and RBV under normal haemodynamic circumstances. Depending on the GA at measurement and the measurement location, the relation between the heart rate and RBV differed. The change in this relation can be explained by limited microcirculatory vasodilation at a lower GA at measurement when compared to a higher GA^42–44^. This phenomenon was observed at all measurement locations. Development of control of vascular tone modulation starts immediately after birth and continues up until approximately the 4th week after gestation^45,46^. Several studies have investigated the effect of postnatal age on blood flow and RBV during the first weeks after birth, yet have found varying results^47–50^. We were unable to find a relation between the postnatal age and the RBV. Most studies agree that, in line with literature on skin development, there are structural changes in the microcirculation after birth. The skin microcirculation of healthy term infants at a postnatal age of approximately 3 months was reported to have a vessel structure similar to that of the adult microcirculation^45^. However, not all studies have found changes in blood flow or RBV within the first weeks after birth^46^, possibly as a result of the variety in applied methods and technologies for measuring these parameters.

An exploratory analysis found no association between PDA and RBV or TBF. This was most likely caused by the low number of included infants with a haemodynamically significant PDA. The current definition of a significant PDA was based on the hospital guideline, which divided PDA severity into medium or severe. To date, there is no international consensus on the definition of PDA. To optimize detection of a potential relation between PDA and the microcirculation, a ductus arteriosus diameter threshold of 2.5 mm as used by Hiedl et al*.* can be considered^51^. However, their results suggest that an altered microcirculation could also be found with a haemodynamically non-significant PDA. The combination of DLS measurements on both extremities with a cardiac ultrasound could provide the information needed to properly assess the association between DLS measurements and a haemodynamically significant PDA.

The effect of phototherapy on DLS measurements showed significantly higher TBF levels in infants with phototherapy, without an increase in RBV. The large wavelength distance between DLS (852.4 nm) and phototherapy (450 nm) suggests that optical interference is not responsible for this effect. A more plausible explanation is vasodilation due to an increased skin temperature, which has also been shown to be present in LED phototherapy^52^. Although no core temperature differences were found, this is not representative of skin temperature that is directly heated by phototherapy.

The study protocol was primarily aimed at the comparison of heart rate measured with electrocardiography and the DLS sensor in stable infants admitted to the NICU. Although the relationship between RBV and the heart rate could be investigated in the entire study population, sub-analyses on other relationships or associations were more difficult to investigate due to a low variation in patient demographics and the absence of longitudinal measurements. Other factors that potentially influence the microcirculation or sensor measurement, such as haemoglobin levels or skin tone, were not included in this study. Similar to other sensor technologies, the main risk in particularly preterm infants is the use of skin adhesives that can cause irritation. As reported previously, one of the most important clinical limitations for the implementation of the DLS sensor in current care is the effect of movement on the measurements, potentially affecting TBF, RBV and relHI values as well^32^. Further iterations of the technology should integrate adequate band-pass filters for heart rate and improve sensor attachment methods. Flow measurements are, however, less dependent on the adequate detection of heartbeats, reducing the influence of mechanical oscillations on the reliability of these measurements.

RelHIs provide information on the shear rates in blood vessels of different sizes. When applying a predefined band pass on the power spectrum of the DLS signal, specific frequencies correspond to specific shear rates. The relHIs form a normalised parameter that is independent of local blood volume, allowing comparison of relHIs over time and between measurement sites^39^. Under pathophysiological circumstances haemodynamic stability and responsiveness of the microcirculation are lost. For example, microcirculatory impairment leads to a change in blood flow and modulation of vessel diameters. The applicability of the predefined relation between the specific frequencies and shear rates is then likely to be influenced. When interpreting changes of the relHIs over time, patient-specific factors affecting the microcirculatory blood flow should be taken into account. Identification of relHIs patterns that are specific for pathophysiological conditions such as oedema, therapeutic hypothermia or sepsis deserves investigation. Whether the cutaneous microcirculation is representative of the splanchnic microcirculation in all of these conditions is not known.

The most distinct separation between relHIs was observed at the lower extremity, providing the optimal site for measuring microcirculatory responses to central changes and the presence of microcirculatory impairment. The IQRs around the median relHIs showed a large variety in the microcirculatory flow distribution between infants, most likely influenced by the environmental temperature and air circulation over the skin. Measurement of the relHIs at the forehead showed large IQRs and a shift towards the larger vessels, as this was the only location in this study population that was not covered. This exposure is likely to have caused a relative vasoconstriction of the small vessels, resulting in a shift in blood flow towards the largest vessels in the microcirculation. The varying spread and distribution of the relHIs between measurement sites can be indicative of local microvascular activity. Other explanations for varying distributions between measurement locations are differences in epidermal thickness or the vasomotor innervation of these locations. The skin microcirculation of the forehead is innervated by both the sympathetic and parasympathetic nervous system, while other locations are only innervated by the sympathetic nervous system^53–55^.

Heart rate decelerations often occur in preterm neonates and have several origins. Vagal stimulation due to immaturity of the autonomic nervous system^56^, immaturity of the centres in the medulla oblongata^57^, and events of apnoea and hypoxemia^58,59^ are suggested as possible origins. The reported changes in relHIs before the onset of a heart rate deceleration reflect a redistribution of the blood flow within the microcirculation. This is potentially an autoregulatory mechanism to counteract central blood pressure changes that follow a decrease in heart rate. The mean perfusion pressure is dependent on both systemic vascular resistance and CO. Prior to a heart rate deceleration, relHIs often showed fast changes that could indicate an increase in systemic vascular resistance to maintain perfusion pressure. In neonatal clinical care the occurrence of bradycardia is associated with the impairment of perfusion with potential consequences depending on frequency and severity^60,61^. Interestingly, we found changes in microcirculatory blood flow to occur regardless of the absolute drop in heart rate and the initial heart rate level at the time of the event. This could mean that transient decelerations such as investigated in this study can have clinical consequences even when the generally used definition of bradycardia is not met. The ability to detect heart rate decelerations in the skin microcirculation suggests that also central organ systems and the brain in particular endure perfusion fluctuations. Although this exploratory study did not validate the investigated new DLS parameters, the study of these parameters for specific clinical indications could contribute to our ability to monitor haemodynamics in the intensive care.

## Conclusions

DLS technology is a non-invasive miniaturized method with the potential of continuously monitoring haemodynamic parameters of the skin microcirculation. This technology should be compared to validated flow measurements in future studies. The most important and currently clinically unavailable parameters are TBF, RBV and relHIs. In haemodynamically stable infants, the lowest value of TBF was recorded at the forehead. RBV values were significantly related to heart rate, GA at measurement and measurement location. Changes in relHIs were recorded substantially earlier than changes in ECG during a heart rate deceleration. Using this technology, the interaction between macrocirculation and microcirculation can be measured. Further evaluation of this technology in different settings, such as in critically ill infants, can prove its clinical value.

## Supplementary Information

Supplementary Information.

## Data Availability

The datasets used and/or analysed during the current study are available from the corresponding author on reasonable request.

## Abbreviations

DLS: Dynamic light scattering
TBF: Total blood flow
RBV: Red blood cell velocity
relHI: Relative haemodynamic index
CO: Cardiac output
GA: Gestational age
NICU: Neonatal intensive care unit
ECG: Electrocardiography
AU: Arbitrary unit
HI: Haemodynamic index
SpO_2_: Oxygen saturation measured with pulse oximetry
PDA: Patent ductus arteriosus
bpm: Beats per minute
AUC: Area under the curve
IQR: Interquartile range
SD: Standard deviation

## References

[1] Bennett VA, Vidouris A, Cecconi M (2018). Effects of fluids on the macro- and microcirculations. Crit. Care.

[2] De Backer D (2013). Microcirculatory alterations in patients with severe sepsis: Impact of time of assessment and relationship with outcome. Crit. Care Med..

[3] Edul VS (2012). Quantitative assessment of the microcirculation in healthy volunteers and in patients with septic shock. Crit. Care Med..

[4] Ince C (2015). Hemodynamic coherence and the rationale for monitoring the microcirculation. Crit. Care.

[5] Kuiper JW, Tibboel D, Ince C (2016). The vulnerable microcirculation in the critically ill pediatric patient. Crit. Care.

[6] Tachon G (2014). Microcirculatory alterations in traumatic hemorrhagic shock. Crit. Care Med..

[7] Trzeciak S (2008). Early increases in microcirculatory perfusion during protocol-directed resuscitation are associated with reduced multi-organ failure at 24 h in patients with sepsis. Intensive Care Med..

[8] van Genderen ME (2014). Microvascular perfusion as a target for fluid resuscitation in experimental circulatory shock. Crit. Care Med..

[9] Abay TY, Kyriacou PA (2015). Reflectance photoplethysmography as noninvasive monitoring of tissue blood perfusion. IEEE Trans. Biomed. Eng..

[10] Lima A, Bakker J (2005). Noninvasive monitoring of peripheral perfusion. Intensive Care Med..

[11] Manorama AA, Baek S, Vorro J, Sikorskii A, Bush TR (2010). Blood perfusion and transcutaneous oxygen level characterizations in human skin with changes in normal and shear loads–implications for pressure ulcer formation. Clin. Biomech..

[12] Cutolo M, Pizzorni C, Sulli A (2005). Capillaroscopy. Best Pract. Res. Clin. Rheumatol..

[13] Groner W (1999). Orthogonal polarization spectral imaging: A new method for study of the microcirculation. Nat. Med..

[14] Hutchings S, Watts S, Kirkman E (2016). The Cytocam video microscope: A new method for visualising the microcirculation using Incident Dark Field technology. Clin. Hemorheol. Microcirc..

[15] Ince C (2005). The microcirculation is the motor of sepsis. Crit. Care.

[16] Micheels J, Alsbjorn B, Sorensen B (1984). Laser doppler flowmetry: A new non-invasive measurement of microcirculation in intensive care?. Resuscitation.

[17] Riva C, Ross B, Benedek GB (1972). Laser Doppler measurements of blood flow in capillary tubes and retinal arteries. Invest. Ophthalmol..

[18] Maitoza LA, Neeman E, Funaro M, Pierce RW (2020). Relevance of microvascular flow assessments in critically ill neonates and children: A systematic review. Pediatr. Crit. Care Med..

[19] den Uil CA (2010). Impaired microcirculation predicts poor outcome of patients with acute myocardial infarction complicated by cardiogenic shock. Eur. Heart J..

[20] Jhanji S, Lee C, Watson D, Hinds C, Pearse RM (2009). Microvascular flow and tissue oxygenation after major abdominal surgery: Association with post-operative complications. Intensive Care Med..

[21] Sakr Y, Dubois MJ, De Backer D, Creteur J, Vincent JL (2004). Persistent microcirculatory alterations are associated with organ failure and death in patients with septic shock. Crit. Care Med..

[22] Scorcella C (2018). MicroDAIMON study: Microcirculatory DAIly MONitoring in critically ill patients: A prospective observational study. Ann. Intensive Care.

[23] Top AP, Ince C, de Meij N, van Dijk M, Tibboel D (2011). Persistent low microcirculatory vessel density in nonsurvivors of sepsis in pediatric intensive care. Crit. Care Med..

[24] Trzeciak S (2007). Early microcirculatory perfusion derangements in patients with severe sepsis and septic shock: Relationship to hemodynamics, oxygen transport, and survival. Ann. Emerg. Med..

[25] van Genderen ME, Lima A, Akkerhuis M, Bakker J, van Bommel J (2012). Persistent peripheral and microcirculatory perfusion alterations after out-of-hospital cardiac arrest are associated with poor survival. Crit. Care Med..

[26] Vellinga NA (2015). International study on microcirculatory shock occurrence in acutely ill patients. Crit. Care Med..

[27] De Backer D, Creteur J, Dubois MJ, Sakr Y, Vincent JL (2004). Microvascular alterations in patients with acute severe heart failure and cardiogenic shock. Am. Heart J..

[28] De Backer D (2006). The effects of dobutamine on microcirculatory alterations in patients with septic shock are independent of its systemic effects. Crit. Care Med..

[29] Buijs EA (2014). Increasing mean arterial blood pressure and heart rate with catecholaminergic drugs does not improve the microcirculation in children with congenital diaphragmatic hernia: A prospective cohort study. Pediatr. Crit. Care Med..

[30] Dubin A (2009). Increasing arterial blood pressure with norepinephrine does not improve microcirculatory blood flow: A prospective study. Crit. Care.

[31] Jhanji S, Stirling S, Patel N, Hinds CJ, Pearse RM (2009). The effect of increasing doses of norepinephrine on tissue oxygenation and microvascular flow in patients with septic shock. Crit. Care Med..

[32] Gangaram-Panday NH (2020). Dynamic light scattering: A new noninvasive technology for neonatal heart rate monitoring. Neonatology.

[33] Broens S (2021). Clinical and Preclinical Optical Diagnostics II.

[34] Wu Z (2016). Postoperative hemodynamic index measurement with miniaturized dynamic light scattering predicts colorectal anastomotic healing. Surg. Innov..

[35] Azhibekov T, Noori S, Soleymani S, Seri I (2014). Transitional cardiovascular physiology and comprehensive hemodynamic monitoring in the neonate: Relevance to research and clinical care. Semin. Fetal. Neonatal. Med..

[36] De Santis P (2021). Incoherence between systemic hemodynamic and microcirculatory response to fluid challenge in critically ill patients. J. Clin. Med..

[37] Ovadia Z (1995). Noninvasive evaluation of microcirculatory hemodynamic changes during hemorrhage followed by saline or blood transfusion. Shock.

[38] IEC. Medical electrical equipment—Part 2–27 Particular requirements for the basic safety and essential performance of electrocardiographic monitoring equipment (IEC 60601-2-27:2011, IDT; IEC 60601-2-27:2011/C1:2012, IDT), 39–40 (2014).

[39] Fine I, Kaminsky AV, Shenkman L (2016). A new sensor for stress measurement based on blood flow fluctuations. Proc. SPIE.

[40] Sakariassen KS, Orning L, Turitto VT (2015). The impact of blood shear rate on arterial thrombus formation. Future Sci. OA.

[41] Pinheiro, J. B. D., DebRoy, S., Sarkar, D., R Core Team. *nlme: Linear and Nonlinear Mixed Effects Models. R package version 3.1–150*, https://CRAN.R-project.org/package=nlme (2020).

[42] Norman M (2008). Low birth weight and the developing vascular tree: A systematic review. Acta Paediatr..

[43] Fluhr JW (2010). Functional skin adaptation in infancy: Almost complete but not fully competent. Exp. Dermatol..

[44] Takayanagi T, Fukuda M, Nakazawa M, Tanaka S, Yoshinaga M (1999). Response of skin blood volume, velocity and flow to local warming in newborns, measured by laser Doppler flowmetry. Pediatr. Int..

[45] Perera P, Kurban AK, Ryan TJ (1970). The development of the cutaneous microvascular system in the newborn. Br. J. Dermatol..

[46] Wu PY (1980). Peripheral blood flow in the neonate; 1 Changes in total, skin, and muscle blood flow with gestational and postnatal age. Pediatr. Res..

[47] van Elteren HA, de Jonge RC, van Rosmalen J, Ince C, Reiss IK (2016). Adaptation of the cutaneous microcirculation in preterm neonates. Microcirculation.

[48] Kroth J (2008). Functional vessel density in the first month of life in preterm neonates. Pediatr. Res..

[49] Jahnukainen T, van Ravenswaaij-Arts C, Jalonen J, Välimäki I (1993). Dynamics of vasomotor thermoregulation of the skin in term and preterm neonates. Early Hum. Dev..

[50] Top AP, van Dijk M, van Velzen JE, Ince C, Tibboel D (2011). Functional capillary density decreases after the first week of life in term neonates. Neonatology.

[51] Hiedl S, Schwepcke A, Weber F, Genzel-Boroviczeny O (2010). Microcirculation in preterm infants: Profound effects of patent ductus arteriosus. J. Pediatr..

[52] Yassin FC (2021). Optimization of the incubator air temperature during LED phototherapy treatment for the preterm infant. Eur. J. Pediatr..

[53] Ishiguro A (2010). Skin and subcutaneous blood flows of very low birth weight infants during the first 3 postnatal days. J. Matern. Fetal Neonatal. Med..

[54] Petrofsky J (2015). Control of Skin Blood Flow.

[55] Toda N, Okamura T (2012). Cerebral blood flow regulation by nitric oxide in Alzheimer's disease. J. Alzheimers Dis..

[56] Church SC, Morgan BC, Oliver TK, Guntheroth WG (1967). Cardiac arrhythmias in premature infants: An indication of autonomic immaturity?. J. Pediatr..

[57] Winter ST, Samueloff M, Cohen NJ, Porges A, Gross E (1966). Neonatal cardiac deceleration on suckle feeding. Am. J. Dis. Child..

[58] Storrs CN (1977). Cardiovascular effects of apnoea in preterm infants. Arch. Dis. Child..

[59] Upton CJ, Milner AD, Stokes GM (1991). Apnoea, bradycardia, and oxygen saturation in preterm infants. Arch. Dis. Child..

[60] Nagraj VP, Sinkin RA, Lake DE, Moorman JR, Fairchild KD (2019). Recovery from bradycardia and desaturation events at 32 weeks corrected age and NICU length of stay: An indicator of physiologic resilience?. Pediatr. Res..

[61] Poets CF (2015). Association between intermittent hypoxemia or bradycardia and late death or disability in extremely preterm infants. JAMA.

