# Supplementary material for: A novel non-invasive method of measuring microcirculatory perfusion and blood velocity in infants: a pilot study
Source: Sci Rep. 2022 May 6;12:7459. doi: 10.1038/s41598-022-10911-8 (PMC9076848; doi:10.1038/s41598-022-10911-8)
Supplement: Supplementary file 1 — Supplementary Information. [file 41598_2022_10911_MOESM1_ESM.pdf]

A novel non-invasive method of measuring microcirculatory perfusion and blood velocity in infants: a pilot study

Norani H. Gangaram-Panday[1], Louwrina H. te Nijenhuis[1], Ilya Fine[2], Irwin K.M. Reiss[1], Willem van Weteringen[1]

[1] Department of Pediatrics, Division of Neonatology, Erasmus MC Sophia Children's Hospital, University Medical Center Rotterdam, Rotterdam, The Netherlands

[2] Elfi-Tech Ltd., Rehovot, Israel

Supplementary information

Supplementary Table S1 TBF and RBV levels at all measurement sites

	Location		Forehead	Upper Ex.	Thorax	Abdomen	Lower Ex.
		Mean (SD)	*p*-value	*p*-value	*p*-value	*p*-value	*p*-value
TBF	Forehead	1139 (449)	-	<0.001	<0.001	<0.001	<0.001
	Upper Ex.	1767 (457)		-	0.0348	0.023	0.786
	Thorax	1860 (523)			-	0.291	0.317
	Abdomen	2099 (682)				-	0.045
	Lower Ex.	1741 (465)					-
RBV	Forehead	1468 (157)	-	0.535	0.710	0.405	0.685
	Upper Ex.	1491 (150)		-	0.870	0.717	0.320
	Thorax	1484 (136)			-	0.407	0.471
	Abdomen	1508 (195)				-	0.439
	Lower Ex.	1452 (134)					-

TBF = total blood flow, RBV = relative blood velocity, Ex. = extremity, SD = standard deviation. TBF is presented in arbitrary units and RBV in Hz^{-1}. Significance was set at a *p*-value <0.005.

Supplementary Table S2 Patient baseline characteristics per phototherapy group

	**Phototherapy (n = 8)**	**No phototherapy (n = 23)**	***p*-value**
GA at birth (weeks)	30 6/7 (30 3/7 – 32 5/7)	28 6/7 (24 6/7 – 40 2/7)	0.067
Birth weight (g)	1585 (1190 – 2840)	1160 (575 – 4000)	**0.015**
SGA	6 (75)	12 (52.2)	0.477
APGAR score at 1 min	5 (4 – 7)	7 (5 – 8)	0.315
APGAR score at 5 min	6 (6 – 8)	8 (7 – 9)	0.055
Arterial umbilical cord pH	7.26 (7.26 – 7.30)	7.31 (7.25 – 7.35)	0.261
Gender (male)	5 (62.5)	10 (43.5)	0.605
Multiple birth	4 (50)	7 (30.4)	0.571
GA at measurement (weeks)	31 4/7 (30 6/7 – 33 4/7)	32 2/7 (28 4/7 – 42 2/7)	0.735
Weight at measurement (g)	1432 (1160 – 2040)	1345 (910 – 4200)	0.557
Postnatal age (days)	3 (3 – 6)	10 (5 – 36)	**0.007**
Admission survival	8 (100)	21 (91.3)	0.979
Ventilation			1.000
Non-invasive	6 (75)	18 (78.3)	
No ventilation	2 (25)	5 (21.7)	
Environment			0.477
Incubator	6 (75)	12 (52.2)	
Heat mattress	2 (25)	11 (47.8)	
Incubator temperature (°C)	30.5 (29.9 – 32.0)	31.0 (29.4 – 32.6)	0.964
Body temperature (°C)	36.7 (36.5 – 37.1)	36.8 (36.5 – 37.2)*	0.741
Heart rate (bpm)	151 (148 – 156)	153 (145 – 166)	0.636

GA at birth, birth weight, GA at measurement and weight at measurement are reported as median (range). All other data is presented as median (IQR) or number (%). GA = gestational age, SGA = small for gestational age. *Missing values (n = 3).

Supplementary Figure S1

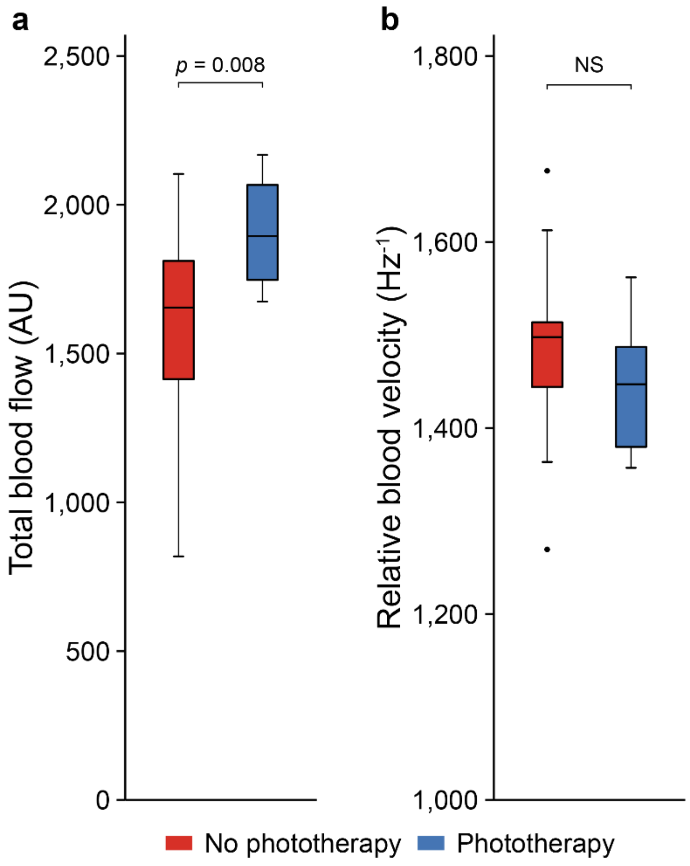

Supplementary Figure S1 Boxplots of DLS parameters perfusion (total blood flow) (**a**) and relative blood velocity (**b**) for phototherapy groups. Significance was found between perfusion measurements when patients received phototherapy vs no phototherapy. AU = arbitrary unit.

Supplementary Table S3 Output linear mixed effects model

n = 31,200	Value	Standard error	t-value	*p*-value
Intercept	1028.38	176.49	5.83	<0.001
Heart rate - 1	474.41	72.66	6.53	<0.001
Heart rate - 2	111.53	134.17	0.83	0.406
Heart rate - 3	-616.31	74.75	-8.25	<0.001
Location - F	68.60	10.92	6.28	<0.001
Location - L	71.86	11.02	6.52	<0.001
Location - T	26.08	10.97	2.38	0.017
Location - U	107.51	10.88	9.88	<0.001
GA at measurement	1.67	0.76	2.20	0.036
Heart rate - Location				
1 - F	-72.32	13.83	-5.23	<0.001
2 - F	-291.15	25.05	-11.62	<0.001
3 - F	-189.46	12.29	-15.41	<0.001
1 - L	-178.25	14.24	-12.52	<0.001
2 - L	-414.80	24.82	-16.71	<0.001
3 - L	-356.98	12.08	-29.55	<0.001
1 - T	-144.27	14.26	-10.12	<0.001
2 - T	-134.72	25.04	-5.38	<0.001
3 - T	-197.98	13.18	-15.03	<0.001
1 - U	-67.83	14.26	-4.76	<0.001
2 - U	-386.00	24.29	-15.89	<0.001
3 - U	-251.39	12.29	-20.46	<0.001
Heart rate - GA at measurement				
1	-1.60	0.31	-5.25	<0.001
2	0.69	0.53	1.30	0.192
3	4.09	0.33	12.52	<0.001

n = number of included data points. F = forehead, L = lower extremity, T = thorax, U = upper extremity, GA = gestational age.
